# Prolonged Hospitalisations in People With Dementia and Comorbid Mental and Behavioural Disorders—A Data Linkage Study

**DOI:** 10.1111/psyg.70156

**Published:** 2026-03-09

**Authors:** Clare Stuart, Kate Jackson, Henry Brodaty, Lee‐Fay Low, Brian Draper

**Affiliations:** ^1^ Mental Health Branch NSW Ministry of Health Sydney Australia; ^2^ Centre for Healthy Brain Ageing (CHeBA), Discipline of Psychiatry & Mental Health, School of Clinical Medicine University of NSW Sydney Australia; ^3^ Faculty of Medicine and Health University of Sydney Sydney Australia; ^4^ Discipline of Psychiatry & Mental Health University of NSW Sydney Australia

**Keywords:** age, BPSD, comorbidity, hospital, length of stay

## Abstract

**Objectives:**

To estimate the prevalence of extreme behavioural and psychological symptoms of dementia (BPSD) in hospitalised patients in comparison with people with dementia with a long hospital stay but no mental health and behavioural problems related to dementia.

**Design:**

Retrospective analysis using the Admitted Patient Data Collection for people aged ≥ 50 with a diagnosis of dementia who had a long stay (defined as ≥ 42 days) in a public hospital between January 2015 and December 2019.

**Setting:**

New South Wales (NSW), Australia.

**Participants:**

People with dementia aged ≥ 50 years with long stays in NSW hospitals.

**Measurements:**

Characteristics of behaviour‐related long stays (BRLS) and non‐behaviour‐related long stays (NBRLS) cohorts were compared using Welch's *t*‐test and Fisher's exact test.

**Results:**

There were 115 people with dementia who had 120 BRLS (range 42–2043 days, median 86 days, IQR 53–151 days) and 6186 people with dementia who had 7523 NBRLS (range 42–5750 days, median 61 days, IQR 49–84 days). Those in the BRLS cohort were younger by a mean of 5.96 years (*p* < 0.001; Welch's *t*‐test, 95% CI 4.18–7.74) and more likely to be men (0.0062, *p* < 0.001, Fisher's exact test). BRLS occurred predominantly in non‐psychiatric public hospitals (92.4%). People with BRLS were more likely to have mental health comorbidities (*n* = 65, 56.5%) than people with NBRLS (2011, 32.7%).

**Conclusions:**

BRLS in people with dementia are less common than NBRLS and are more likely to occur in the context of mental health comorbidities and in younger males.

## 1 Introduction

People living with dementia are regularly admitted to acute hospitals with hospitalisation rates more than double those of people without dementia [1]. Admissions are more likely to be emergency cases, with pneumonia, urinary tract infections, injuries and neurological disorders being common causes [2, 3, 4].

People with dementia have longer hospital stays than people without dementia for a range of medical, behavioural, social and organisational reasons [5]. One key reason is that people with dementia are more prone to delirium, which may be present on admission or develop during admission [6, 7, 8, 9]. Hospitalisations due to injuries including fractures, traumatic brain injuries, burns and poisoning have longer lengths of stay (LOS) in people with dementia [10]. Other hospital‐acquired conditions such as pneumonia, urinary tract infections and pressure sores are also associated with longer LOS in people with dementia [11]. There may be a vicious circle where these medical conditions and injuries may worsen cognition and functional decline that in turn increases LOS [5]. Non‐medical factors that influence LOS include organisational issues within the hospital [12], being under the care of a consultant with limited dementia experience [13], delays in discharge to long term residential care or receipt of community services [12] and not having the financial resources to afford services [13].

Behavioural and psychological symptoms of dementia (BPSD) such as aggression, agitation, psychosis and depression also increase LOS in people with dementia and predict transfer to residential aged care [14]. In those who develop delirium in hospital, it is the associated behavioural disturbances that are the proximal cause of the prolonged LOS [7]. For people with dementia admitted to hospital due to BPSD, longer hospitalisations occur in males with aggressive behaviour and when there are features of psychosis [15, 16]. While treatments of aggressive behaviour in dementia are effective, the onset of treatment effect may take some weeks with limited treatment response in some individuals [17]. Furthermore, when sufficient improvement occurs for hospital discharge, increased support for family carers or transfer to a residential facility may be required, which may result in delayed discharge [15].

The current study is part of a New South Wales (NSW) Health project examining ‘extreme BPSD’, the top tier of a seven‐tiered model of management of BPSD originally proposed by our team and adopted by the Australian Commonwealth and some State governments [18]. In earlier phases of the project, interviews with staff from older people's mental health and geriatric medical services as well as family and other carers indicated that unpredictable aggression was a key feature. The individuals were more likely to be younger males, often with comorbid mental illness and complex needs. Based on a combination of anecdotal reports and data collected by the NSW Ministry of Health, it was estimated that there were between 30 and 60 individuals in NSW experiencing extreme BPSD at any one time and that they were likely to have prolonged hospital admissions of between 3 and 12 months [19]. This is much longer than the mean LOS of 16.5 days that people with dementia have in NSW hospitals [4]. There is a lack of published data on the prevalence of extreme BPSD in acute hospitals to inform policy and service planning.

The current study explores prolonged hospitalisations of people with dementia and comorbid mental health and behavioural problems in NSW. The aims of the study are to estimate the prevalence of extreme BPSD in hospitalised patients with dementia and to develop a profile comprising sociodemographics, clinical features, comorbidities, hospital acquired conditions and patterns of health service use in comparison with people with dementia with a long hospital stay but no recorded mental health and behavioural problems related to dementia.

## 2 Method

A retrospective analysis was conducted of administrative data for people aged ≥ 50 with a diagnosis of dementia who had a long stay (defined as ≥ 42 days the average LOS in NSW older persons' acute mental health wards in 2017–18) [20] in a NSW hospital between January 2015 and December 2019. Ethical approval was obtained from the NSW Population and Health Services Research Ethics Committee.

### 2.1 Datasets and Data Linkage

The Admitted Patient Data Collection (APDC) was the primary dataset. The APDC includes information on all inpatient admissions from all public and private general hospitals and public psychiatric hospitals in NSW; however, length of stay data for private hospitals is not available. Within the ADPC, hospital stays are made up of one or more episodes of care. A new episode is created for every change in care type within the hospital and each transfer between hospitals. Diagnoses were classified using the International Classification of Diseases, 10th Revision, Australian Modification (ICD‐10‐AM) [21]. Each episode of care can have up to 55 diagnoses recorded, including one primary diagnosis.

People meeting inclusion criteria in the APDC were probabilistically linked to records in the NSW Emergency Department Data Collection using *ChoiceMaker* [22]. This process uses identifying information to allow linked records for the same individual to be identified and extracted. The false positive rate for this process is 5/1000 records (0.5%) [23].

### 2.2 Inclusion Criteria

As there are no codes in the APDC to indicate extreme BPSD, long stays in people aged ≥ 50 identified as having dementia in combination with mental health or behavioural codes were used as a proxy measure. A dementia diagnosis was identified using the following codes and associated subcodes: ‘Dementia in Alzheimer's disease’ (F00), ‘Vascular dementia’ (F01), ‘Dementia in other diseases classified elsewhere’ (F02), ‘Unspecified dementia’ (F03), ‘Delirium superimposed on dementia’ (F05.1) or ‘Alzheimer's disease’ (G30) before, during or within 12 months of the long–stay (Table 1). ‘Mental and behavioural disorders due to use of alcohol, residual and late onset psychotic disorders’ (F10.7) was also included because this diagnostic code can be used for alcohol‐related dementia.

**TABLE 1:** People with dementia with prolonged hospital stays (42+ days) in NSW hospitals 2015–2019.

	With behaviour related long stays			Non‐behaviour related long stays			Behaviour related long stays compared to non‐behaviour related long stays
	With mental health diagnosis	Without other mental health diagnosis	Total with behaviour related long stays	With mental health diagnosis	Without other mental health diagnosis	Total with non‐behaviour related long stays	
People with long stays	65	50	115	2011	4131	6142	
Mean age	71.47	73.23	73.02	74.59	81.53	78.98	*p* < 0.0001 Unpaired *t*‐test, 95% CI 4.17–7.74
% (*n*) Males	66.15% (43)	60.00% (30)	63.48% (73)	52.86% (1063)	49.48% (2044)	50.59% (3107)	*p* < 0.01; 0.0062 Fisher's exact test
% (*n*) Married	33.85% (22)	50.00% (25)	40.87% (47)	38.99% (784)	47.64% (1968)	44.81% (2752)	n.s.
% (*n*) Born in non‐English speaking countries^a^	23.08% (15)	36.00% (18)	28.70% (33)	28.34% (570)	27.77% (1147)	27.96% (1717)	n.s.
% With regional or rural local health district of residence	36.92% (24)	34.00% (17)	35.65% (41)	34.01% (684)	32.68% (1350)	33.13% (2034)	n.s.
Number of long stays^b^	69	51	120	2522	4971	7523	—
Length of stay (days)							
Mean	130.52	224.15	170.82	121.75	77.83	92.44	n.s.
Median	86	87	86	70	57	61	—
Range	42–669	42–2043	42–2043	42–5750	42–3672	42–5750	
Inter‐quartile range	52–174	54–130	53–151	53–104	48–77	49–85	

^a^ Non‐English‐speaking countries of birth comprised those other than the main English‐speaking countries of New Zealand, United Kingdom, Ireland, United States of America, Canada and South Africa.

^b^ The number of long stays is greater than the number of people due to some having multiple long stay.

### 2.3 Exclusion Criteria

The cohort excluded: long stays in hospitals within NSW Justice Health facilities; long stays in aged care facilities, and where a long stay preceded a dementia diagnosis by more than 12 months.

### 2.4 Defining Behaviour and Non‐Behavioural Related Long Stay Cohorts

Behaviour‐related long stay (BRLS) was defined as a hospital stay of 42 days or longer in a person with dementia, within which at least one episode of care had an additional diagnosis code of ‘Other mental disorders due to brain damage and dysfunction and to physical disease’ (F06 including all subcodes) and/or ‘Personality and behavioural disorders due to brain disease, damage and dysfunction’ (F07 including any subcodes). The remaining people with dementia with long stays were categorised as having non‐behaviour‐related long stays (NBRLS).

### 2.5 Coding of Mental Health Diagnoses

Mental health comorbidities were defined by the ICD‐AM codes F09 to F99 inclusive, excluding dementia (as defined above), delirium and behaviour (as defined above). BRLS and NBRLS cohorts were further analysed based on whether the person had a long stay with a mental health comorbidity.

### 2.6 Coding of Dementia Type

Types of dementia diagnosed could vary between hospital stays and episodes of care within a stay. If a type of dementia was specified, we used the following hierarchy on the diagnosis used from hospital stays: first, their BRLS; second, their most recent episode of care prior to their BRLS; third, an episode of care in the 12 months following their BRLS. If there was more than one type of dementia diagnosis within a hospital stay but in a different episode of care, the most recent was used. If multiple dementia diagnoses were present in the episode of care, the primary diagnosis was used.

### 2.7 Coding of Hospital Acquired Conditions

We analysed all diagnostic codes that were reported as acquired during the hospital stay, which were grouped based on type of condition and/or organ system involved as well as conditions known to be associated with behaviour change such as falls, injuries, pain syndromes and effects of psychotropic medication.

### 2.8 Strokes

Strokes were defined by the ICD‐AM codes I60 to I64 inclusive. Stroke sequelae were defined as ICD‐AM code I69.

### 2.9 Referrals Into Care

We analysed the referral code for each episode of care within a BRLS. We excluded those that were ‘Type Change Admission’, as these are used when care type changes within a single hospital stay. The Emergency Department Data Collection linked data were used to understand the source of referrals through Emergency Departments (ED) and to analyse the use of EDs.

### 2.10 Data Management and Analysis

Microsoft Excel 2013 and IBM SPSS Statistics 25 software were used. We used a variety of statistical tests to compare characteristics of the BRLS cohort with the NBRLS cohort. Average age of the two cohorts was compared using Welch's *t*‐test. Other characteristics were compared using Fisher's exact test. An independent *t*‐test was run to compare the average number of mental health diagnoses in the episodes of care of the BRLS cohort and the episodes of care of the NBRLS cohort. To test whether mental health or non‐organic psychosis diagnosis was associated with cohort after controlling for age and sex, a logistic regression was conducted with cohort as the dependent variable and where age and sex were entered as covariates, along with any non‐organic psychosis diagnosis and any mental health diagnosis. A post hoc power calculation using g*power showed that with alpha set at 0.5 two tailed, there was a 78% power to detect a small difference (*d* = 0.25) between groups and 99.9% power to detect a medium difference (*d* = 0.5) between the means of the two groups [24].

## 3 Results

In the 5 years between 2015 and 2019, there were 115 people in the BRLS cohort who had 120 long stays that comprised 230 separate episodes of care with LOS ranging from 42 days to 2043 days (5.6 years), (mean 170.8 days, SD 324.9 days, median 86 days, (IQR 53–151) days).

In the NBRLS cohort there were 6186 people who had 7523 long stays with LOS ranging from 42 to 5750 days (15 years) (mean 92.4 days, SD 186.3 days, median LOS 61 days, IQR 49–84).

Of the longest stays (37 > 5 years), 31 (84%) were in public psychiatric hospitals predominantly with a primary psychosis (schizophrenia, schizoaffective disorder, delusional disorder) diagnosis, five (14%) were in general hospitals and one (3%) was in a small rural public service offering both aged care and health services. Those in the BRLS cohort were younger by a mean of 5.96 years (*p* < 0.001; Welch's *t*‐test, 95% CI 4.18–7.74) and more likely to be men (0.0062, *p* < 0.001, Fisher's exact test). Per calendar year, 23 people with dementia on average experienced a BRLS and 1241 people with dementia on average experienced a NBRLS. (see Table 1).

Almost half of those with BRLS were referred through an ED (*n* = 55, 45.8%), with a further 34 (28.3%) being transferred from another hospital, six of which were private hospitals. Emergency Department attendances between 2015 and 2019 are presented in Table 2. The most common sources of referral into EDs for the BRLS cohort were self, family, friends (71.37%, *n* = 703) and residential aged care facility (RACF) (16.35%, *n* = 161). The BRLS cohort had more visits to ED than the NBRLS cohort (*t*(6299) = 2.1663, *p* = 0.0303).

**TABLE 2:** Emergency department presentations behaviour and non‐behaviour‐related long stays 2015–2019.

	Behaviour‐related long stays *N* = 115	Non‐behaviour‐related long stays *N* = 6186
Number (%) at least one visit	112 (97.4%)	5892 (95.3%)
Mean (SD)	8.6 (SD = 8.1)	6.7 (SD = 9.4)
Median	6	5
Range	0–42	0–309
Inter‐quartile range	3–12	3–8

### 3.1 Where Care Was Provided

Most BRLS were in public non‐psychiatric hospitals (90.4%, 12 546 bed days), with a smaller number in public psychiatric hospitals (7.0%, 2290 bed days). Data were missing for 6 stays (2.6%, 334 bed days). Nineteen people with dementia (15.8%) needed to travel out of their own area for their BRLS. For NBRLS, 1205 people with dementia (19.5%) had to travel out of their own area for their NBRLS. Around half of BRLS was provided in metropolitan areas (49% of bed days) and around half in non‐metro areas (49% of bed days); data not available for 2% of bed days. Most care was identified as non‐mental health related (70.9% care episodes, 59.2% bed days); mainly acute care (45.7% care episodes, 28.5% bed days), rehabilitation care (10.4% care episodes, 6.2% bed days) and maintenance care (9.1% care episodes, 22.1% bed days). Mental health related care accounted for 29.1% of care episodes and 40.8% of bed days with some receiving psychogeriatric care (5.7% care episodes, 7.3% bed days).

### 3.2 Principal Diagnosis for BRLS

The most common principal diagnoses for BRLS were ‘dementia’ (35 episodes of care, 3414 bed days [22.5%]), ‘behaviour and/or mental disorders due to brain damage, physical health or alcohol’ (32 episodes of care, 2664 bed days [17.6%]), ‘specific mental disorders’ (30 episodes of care, 2324 bed days, [15.3%]; schizophrenia and schizoaffective disorders were most common) and ‘other disorders of the central nervous system’ (21 episodes of care, 949 bed days (6.3%); delirium and epilepsy were most common). In 18 episodes of care that comprised 3160 bed days (20.8%) the principal diagnosis was listed as ‘problems related to medical facilities and other health care’, a category used when a person remains in hospital due to there not being a more appropriate setting, such as a RACF, available for them. For the NBRLS, the most common principal diagnoses were dementia (2791 episodes of care, 152 475 bed days, [17.8%]), awaiting transfer to residential care (2214 episodes of care, 96 915 bed days [11.3%]), fractures and other injuries (e.g., burns, wounds, dislocations) (1872 episodes, 62 377 bed days [7.3%]), ‘specific mental disorders (1243 episodes, 140 173 bed days, [16.3%]), and other disorders of the central nervous system (1082 episodes, 22 835 bed days, [2.7%]).

### 3.3 Dementia Type for BRLS Cohort

The dementia diagnoses in the BRLS cohort are shown in Table 3. Of the 10 people with a F10.7 and no other dementia diagnosis on their BRLS, three went on to receive another dementia diagnosis within the period of the study. This group of 10 had a lower mean LOS (77.3 days); younger age (65.8 years) and were more likely to be male (90.0%). They had similar rates of mental health comorbidities as the wider BRLS cohort.

**TABLE 3:** Dementia diagnosis of BRLS cohort.

Code	Description	*n*	%
F01	Vascular dementia	44	38%
F03	Unspecified dementia	25	22%
F00/G30	Dementia in Alzheimer's disease (G30.±)	15	13%
F10.7	Mental and behavioural disorders due to use of alcohol, residual and late‐onset psychotic disorder	10	9%
F02.0	Dementia in Pick's disease (G31.0+)	6	5%
F05.1	Delirium superimposed on dementia	5	4%
F02.3	Dementia in Parkinson's disease (G20+)	4	3%
F02.2	Dementia in Huntington's disease (G10+)	2	2%
F02.8	Dementia in other specified diseases classified elsewhere	2	2%
F02.4	Dementia in human immunodeficiency virus [HIV] disease (B22+)	1	1%
n/a	Dementia in Alzheimer's disease (G30.±) and Vascular dementia (F01)	1	1%
Total		115	100%

### 3.4 Mental Health Comorbidity

Within the BRLS cohort there were 65 (56.5%) people with dementia with mental health comorbidity, while in the NBRLS cohort, there were 2011 (32.7%) with mental health comorbidity. The logistic regression model examining the association of mental health comorbidity with the BRLS cohort was statistically significant, chi‐square = 246.24, *p* < 0.001. The model explained 8.5% (Nagelkerke R2) of the variance. People with non‐organic psychosis and with mental health diagnosis were more likely to be in the BRLS rather than the NBRLS group. (see Table 4).

**TABLE 4:** Logistic regression table looking at mental health and non‐organic psychosis association with behaviour related long stay (coded 1) or non‐behaviour related long stay (coded 0).

Effect	Estimate	SE	OR	95% CI lower	95% CI upper	*p*
Intercept	1.427	0.456	4.166			0.002
Sex (male 1, female 2)	−0.233	0.115	0.792	0.632	0.992	0.43
Age	−0.065	0.006	0.937	0.926	0.947	< 0.001
Any non‐organic psychosis (1 = yes, 0 = no)	0.377	0.158	1.458	1.070	1.988	0.017
Any mental health diagnosis (1 = yes, 0 = no)	0.373	0.134	1.452	1.1117	1.889	0.005

Those with mental health comorbidity had a mean LOS longer by 43.3 days (*p* < 0.001, unpaired *t*‐test, 95% CI 42.9–49.7 days) than those without mental health comorbidities. This group was also less likely to be married (*p* < 0.001, Fisher's exact test) and more likely to be male (*p* = 0.013, Fisher's exact test) than those without mental health co‐morbidities.

When people with mental health comorbidities were excluded from both cohorts, those with a BRLS had a mean LOS longer by 62.4 days (*p* < 0.0001, unpaired *t*‐test, CI 30.85–94.02 days) and were also younger by 7.34 years than those with a NBRLS (*p* < 0.0001, unpaired *t*‐test, 95% CI 5.16–9.51 years). The groups did not differ by sex (*p* = 0.112, Fisher's exact test). Episodes of care in the BRLS cohort were significantly more likely to have at least one mental health diagnosis (Chi Squared (8, *N* = 13 813) = 114.024, *p* < 0.001) and to have significantly more mental health diagnoses (*M* = 0.81, SD = 0.93) than the NBRLS cohort (*M* = 0.43, SD = 0.82); *t*(13 811) = 8.50, *p* < 0.001.

### 3.5 Hospital Acquired Conditions

Sixty‐three (54.8%) people in the BRLS cohort had one or more hospital acquired conditions, with the most common being cardiorespiratory disorders (*n* = 31, 27.0%), falls (*n* = 26, 22.6%), and sepsis (*n* = 24, 20.9%). Fifteen people (13.0%) had a head injury, 14 (12.2%) a cognitive/behavioural disorder, and 11 (9.6%) had complications of procedures and treatments including five due to psychotropic drugs. Pain syndromes were noted in nine (7.8%) people (see Table 5).

**TABLE 5:** Prevalence of the most common types of hospital acquired conditions based on number of people who experienced them at least once in their long behaviour stays.

Hospital acquired condition groupings	*n*	%
Cardiorespiratory disorders	31	27.0%
Falls	26	22.6%
All injuries	26	22.6%
Head injuries	15	13.0%
Sepsis	24	20.9%
Metabolic disorders	16	13.9%
Haemodynamic disorders	16	13.9%
Cognitive & behavioural disorders	14	12.2%
Gastro‐intestinal disorders	12	10.4%
Complications of procedures and treatments	11	9.6%
Psychotropic adverse effects	5	4.4%
Pain syndromes	9	7.8%
Musculoskeletal disorders	8	7.0%
Urinary tract disorders (excluding infections)	7	6.1%

### 3.6 Stroke

Out of the 115 people in the BRLS cohort, one patient experienced a stroke during their BRLS. A further four people had stroke as the principal diagnosis on their BRLS. Overall, 15 of the 115 remaining BRLS suggested the person had a history of stroke (13.0%). Out of these, six involved an acute stroke; nine of these involved stroke sequelae. Nine of these people also had a diagnosis of vascular dementia (60%). A further 10 people experienced a stroke only after a BRLS. One person experienced a stroke between two BRLS.

## 4 Discussion

This study provides the first estimate of the prevalence and characteristics of BRLS in people with dementia in NSW hospitals, a jurisdiction in which long term residential care of BPSD is undertaken primarily in RACFs rather than hospitals. In that context, BRLS in people with dementia include those with extreme BPSD unmanageable in RACFs. On average, there were 23 long stays per year, which equates to 0.02% of people with dementia in NSW [1], and is slightly lower than the number of extreme BPSD cases (30–60) estimated in the previous phase of this work [19]. The difference in the estimates may be because the parameters chosen to enumerate possible extreme BPSD (long hospital stay and behaviour‐related diagnostic code) and dementia diagnosis might not accurately capture extreme BPSD cases due to factors such as resolution in less than 6 weeks and failure to code a behavioural disorder.

When compared with NBRLS, which were over 50 times more common equating to 1.1% of people with dementia [1], the people with BRLS were younger, more likely to be male, and more likely to have a comorbid mental health diagnosis, particularly a psychosis such as schizophrenia or schizoaffective disorder. These characteristics were also identified in the earlier qualitative component of the extreme BPSD project [19]. The extent to which the comorbid non‐organic psychoses were responsible for the long stays through treatment‐resistant psychosis rather than behaviour related to the onset of dementia is not known. People with schizophrenia and other psychotic disorders have approximately two to five times the rate of dementia found in the general population, and this is more likely to occur at an earlier age in their late 60s [25, 26]. It is likely that the presence of non‐organic psychosis impacted on discharges to RACFs through reluctance of either the Aged Care Assessment Teams to approve admissions or individual RACFs to accept admissions.

Aggression was the main behaviour of concern identified in the qualitative extreme BPSD project [19]. One of the limitations of hospital coding using ICD10‐AM is the absence of codes for specific behaviours and thus we are unable to determine the precise behavioural issues that contributed to the long hospital stays [21]. In addition, while in most cases the principal diagnosis was determined to be dementia, a behavioural disorder or a mental disorder, in over 20% of cases the principal diagnosis was coded as ‘problems related to medical facilities and other health care’. In the context of BPSD and given that most referrals to ED were from either family and friends or RACFs, the latter ‘diagnosis’ might indicate that RACFs were refusing to accept the patient's admission possibly due to the behavioural history thus prolonging the hospital admission beyond what clinicians felt required hospital care. Similarly, delays in obtaining adequate community aged care services to support families may account for this.

Although dementia and mental disorders were also prominent principal diagnoses in the NBRLS cohort, there were some differences, with fractures and other injuries being common. This is consistent with previous studies that have found that LOS in patients with hip fractures or burns is significantly longer in those with dementia [27, 28].

The other principal diagnosis prominent in the NBRLS cohort, ‘waiting admission to residential service’ reflects unavailability of placement options rather than problems in placement due to the nature of the patient's condition.

Non‐Alzheimer dementias predominated with vascular dementia (38%) being the most prevalent, much higher than found in an earlier study of dementia in NSW hospitals [4]. Strokes were common before and after the BRLS and this was likely the reason for the high prevalence of vascular dementia. Only around 6% of bed days were for rehabilitation thus it is unlikely that stroke‐related physical impairment was a major factor in the BRLS. Hospital‐acquired conditions occurred in over half of the BRLSs, notably cardiorespiratory disorders, falls, sepsis and head injuries. Previous studies have shown that people with dementia have a higher risk of hospital‐acquired delirium, falls, pressure injury and pneumonia [29], potentially preventable conditions that are associated with longer LOS and higher costs [9, 11].

Overall, the factors contributing to the BRLS appear to be a complex mix of mental health issues, behavioural concerns, physical comorbidity, hospital acquired conditions and discharge delays related to finding residential facilities or community care able and willing to accept the transfer. These findings are consistent with those from the qualitative extreme BPSD project [19]. This suggests that there is no single solution to changing clinical practice. Service delivery requires interdisciplinary care involving geriatricians and old age psychiatrists as well as nursing and a range of allied health staff. Social workers are required to address the needs of families and facilitate discharges, while specialist input to RACFs and community settings post‐discharge is required to support care staff and families by addressing ongoing and emerging behavioural concerns. Some outcomes of the extreme BPSD project included the establishment of a statewide extreme BPSD older persons' mental health consultation/liaison service pilot, the release of design guidance for improving general hospital environments for people with severe‐extreme BPSD and the implementation of training strategies to improve general hospital staff skills in managing BPSD and a revision of the BPSD Handbook for NSW Health clinicians [30, 31].

There are some limitations to this study. We have used prolonged LOS in people with dementia and an associated F06 or F07 diagnosis as a proxy for extreme BPSD as there is no specific hospital coding for BPSD. Despite the similarity of the clinical features of the BRLS cohort with those found in the qualitative extreme BPSD project, the extent to which the combination of a dementia diagnosis with a F06 or F07 diagnosis is consistent with BPSD is unclear and there are likely to be cases with LOS of less than the six‐week cut off we selected. There may have been differential mortality with extreme BPSD possibly more likely to die within 42 days. Hospital coding is known to underestimate dementia cases and so some will have been missed this way. We included the diagnostic code F10.7 (residual and late onset psychotic disorders related to alcohol) as cases of alcohol‐related dementia might attract this code but concede that some might not have had dementia. Length of stay data were missing relating to private hospital stays, meaning no stays that were entirely in private hospitals could be identified as long and where there were stays involving episodes of care in both private and public hospitals, long stays were only able to be identified if the public hospital component was a long stay. There are few private hospitals in NSW that focus on complex geriatric care with most transferring such cases to public hospitals as noted in six cases in this dataset. However, it is possible that there were some cases with prolonged LOS related to dementia that were managed entirely within private hospitals. The dataset has little information about the discharge destination.

In conclusion, this study provides some insights into the complexity of factors contributing to BRLS in people with dementia. Future work needs to improve the identification of BPSD in hospital coding and through other means to get a better understanding of the extent to which BPSD contributes to prolonged LOS in hospitals.

## Funding

The NSW Ministry of Health made data available for this project and funded the data linkage.

## Conflicts of Interest

Clare Stuart reports financial support, administrative support and statistical analysis were provided by NSW Ministry of Health. Brian Draper reports a relationship with Commonwealth Department of Health & Ageing Special Dementia Care Program that includes: consulting or advisory. Henry Brodaty reports a relationship with Eisai, Eli Lilly, Medicines Australia, Roche and Skin2Neuron that includes: board membership. Henry Brodaty reports a relationship with National Health and Medical Research Council that includes: funding grants. Lee‐Fay Low reports a relationship with HammondCare that includes: funding grants. Lee‐Fay Low reports a relationship with The Whiddon Group that includes: funding grants. Lee‐Fay Low reports a relationship with National Health and Medical Research Council that includes: funding grants. Lee‐Fay Low reports a relationship with Australian Government Medical Research Future Fund that includes: funding grants. Lee‐Fay Low reports a relationship with Roche that includes: speaking and lecture fees. Lee‐Fay Low reports a relationship with Dementia Australia NSW that includes: funding grants. Kate Jackson reports a relationship with NSW Ministry of Health that includes: employment. If there are other authors, they declare that they have no known competing financial interests or personal relationships that could have appeared to influence the work reported in this paper.

## Data Availability

The data that support the findings of this study are available on request from the corresponding author. The data are not publicly available due to privacy or ethical restrictions.
